# Flowering time adaption in Swedish landrace pea (*Pisum sativum* L.)

**DOI:** 10.1186/s12863-016-0424-z

**Published:** 2016-08-12

**Authors:** Tytti Vanhala, Kjersti R. Normann, Maria Lundström, James L. Weller, Matti W. Leino, Jenny Hagenblad

**Affiliations:** 1 IFM-Biology, Linköping University, SE-581 83 Linköping, Sweden; 2 Department of Biology, Norwegian University of Science and Technology, Trondheim, Norway; 3 School of Biological Sciences, University of Tasmania, Hobart, TAS 7001 Australia; 4 Nordiska museet - Swedish Museum of Cultural History, SE-643 98 Julita, Sweden

**Keywords:** Crop evolution, *HIGH RESPONSE TO PHOTOPERIOD* (*HR*), *LATE FLOWERING* (*LF*), Legumes, Local adaptation, *STERILE NODES* (*SN*)

## Abstract

**Background:**

Cultivated crops have repeatedly faced new climatic conditions while spreading from their site of origin. In Sweden, at the northernmost fringe of Europe, extreme conditions with temperature-limited growth seasons and long days require specific adaptation. Pea (*Pisum sativum* L.) has been cultivated in Sweden for millennia, allowing for adaptation to the local environmental conditions to develop. To study such adaptation, 15 Swedish pea landraces were chosen alongside nine European landraces, seven cultivars and three wild accessions. Number of days to flowering (DTF) and other traits were measured and the diversity of the flowering time genes *HIGH RESPONSE TO PHOTOPERIOD* (*HR*), *LATE FLOWERING* (*LF*) and *STERILE NODES* (*SN*) was assessed. Furthermore, the expression profiles of *LF* and *SN* were obtained.

**Results:**

DTF was positively correlated with the length of growing season at the site of origin (GSO) of the Swedish landraces. Alleles at the *HR* locus were significantly associated with DTF with an average difference of 15.43 days between the two detected haplotypes. *LF* expression was found to have a significant effect on DTF when analysed on its own, but not when *HR* haplotype was added to the model. *HR* haplotype and GSO together explained the most of the detected variation in DTF (49.6 %).

**Conclusions:**

We show local adaptation of DTF, primarily in the northernmost accessions, and links between genetic diversity and diversity in DTF. The links between GSO and genetic diversity of the genes are less clear-cut and flowering time adaptation seems to have a complex genetic background.

**Electronic supplementary material:**

The online version of this article (doi:10.1186/s12863-016-0424-z) contains supplementary material, which is available to authorized users.

## Background

Pea (*Pisum sativum* L.) was the first genetic model species, used to demonstrate central genetic concepts such as dominance, segregation and independent assortment [1]. It is also a widely cultivated crop species, and a major source of plant protein for both animal and human consumption (FAOSTAT, http://faostat3.fao.org). Pea was most likely domesticated from *Pisum elatius* and spread from the Fertile Crescent in two distinct lineages, eastwards across Southern Asia and westwards over North Africa and the Mediterranean [2]. Archaeological remains suggest a rapid spread across the Mediterranean followed by a marked delay before it began to expand northwards [3, 4]. It has been suggested that this lag in spread was necessary to allow for the evolution of suitable responses to novel light and temperature conditions [5].

The evolution of environmental adaptation must have been a necessity as pea cultivation gradually expanded north. One of the northernmost reaches of pea cultivation is in Sweden where it has been an important crop from Neolithic times and onwards [6]. Different types of pea have been cultivated across the country all the way from the southern tip of Sweden (below the 56^th^ latitude) to near the polar circle (64^th^ latitude) [7]. During the 19^th^ century, pea was cultivated on more than 3 % of the Swedish farmland and was, along cereals and potatoes, one of the most important crop species [8]. Even though pea cultivation declined during the last century, it remained a field crop where large-scale cultivation of landraces (highly variable and locally adapted varieties lacking formal improvement) was actively maintained until the 1950s [9, 10]. Nowadays pea landraces are only cultivated on a small scale as heirloom crops [8].

The parts of Sweden where pea has been cultivated offer a range of climatic conditions with the length of growing season ranging from approximately 140 to 220 days. From previous studies of neutral genetic markers it is clear that Swedish landraces, both past and present, are often very variable and that there is high genetic differentiation among accessions from different parts of Sweden [11, 12]. It is therefore likely that candidate adaptation genes, for example those involved in the timing of flowering, will show signs of adaptation to the climatic differences in different parts of Sweden.

The genetic regulation of the transition to flowering is well known from numerous studies in Arabidopsis and other plants [13]. Corresponding genes have been identified in pea (for a review see [14]). In this study we chose to focus on three genes with well-described effects on flowering time in pea: *HIGH RESPONSE TO PHOTOPERIOD* (*HR* [15]), *LATE FLOWERING* (*LF* [16]) and *STERILE NODES* (*SN* [17]).

At the *HR* locus, the dominant *HR* allele is known to repress flowering under short day (SD) conditions making long day (LD) a requirement for flowering. In spring, during SD conditions, *hr* allows early flowering [14]. It has been shown that *HR* affects flowering time through the circadian clock where it interacts with the circadian clock gene *SN* [15]. *SN* is the pea ortholog of the *LUX ARRHYTHMO* (*LUX*) transcription factor in Arabidopsis [17]. In Arabidopsis *LUX* is a sequence specific DNA-binding protein, which self regulates by binding to its own promoter [18]. It forms an evening complex together with *ELF3* (the ortholog for *HR* in pea) and *ELF4* proteins and this complex, with its *LUX* binding site, regulates the diurnal hypocotyl growth as well as directly regulating the morning clock gene *PRR9* [19]. Under SD, *SN* has been shown to influence flowering time in pea through mutations accelerating the transition to flowering [17, 20]. *LF* produces a *TFL1* homologue that regulates transcription factor activity in the shoot apex, and it was the first pea flowering time locus to be identified at the molecular level [16]. Flowering time variation caused by allelic variation at *LF* is not always associated with mutations in the gene sequence. Instead the expression of *LF* is strongly correlated with flowering time [16]. The *HR* and *SN* act in the leaf as described above while *LF* acts in the shoot apex in the flowering model [14].

Although the molecular functioning of flowering time genes is well known, there are few studies connecting local adaptation to variation in sequence and gene expression of adaptation genes, in particular in crop species (but see [21, 22]). Here we investigate how landraces of pea have adapted to differences in climatic conditions in Sweden. More specifically we have studied a) the effect of genetic diversity on flowering time in pea, b) whether there are signs of flowering time being adapted to different environmental conditions among Swedish landraces, c) what variation in sequence and gene expression of flowering time genes is present in the Swedish landraces, d) how Swedish landraces differ from European landraces, key cultivars and wild pea, in flowering time and production traits.

## Methods

### Plant material and cultivation

In spite of the late abandonment of landrace pea as a field crop in Sweden, only a limited number of accessions with a reliable provenance are preserved at the Nordic Genetic Resource Center. Among these, fifteen Swedish landraces, originating from a range of different cultivation conditions across Sweden, were chosen for the study (Table 1, Additional file 1). In addition, landraces from other parts of Europe (nine accessions), modern cultivars, primarily from Sweden (seven accessions), wild peas (three accessions) and two standard lines, JIC1228 (*Lf d*) and JIC1233 (*lf e*) for late and early flowering respectively [20, 23] were included. The wild peas were all recorded as *P. sativum* var *elatius*, the species suggested as the ancestral species of domesticated pea in Europe [2]*.*

**Table 1:** Accessions included in the study and their genetic and phenotypic diversity in flowering time genes

Accession number	Name	Accession type	Days to flowering^a^	HR genotype	LF haplotype	LF expression^b^	SN haplotype	SN expression^b^
NGB101819	WBH1819 (Norra Rörum)	Swedish landrace	42.2 (1.10)	hr	1	7.28	1	8.72
NGB14154	Maglaby	Swedish landrace	79.4 (10.85)	HR	1	5.92	1	9.00
NGB17873	Puggor från Glimåkra	Swedish landrace	64.2 (2.95)	HR	3	5.84	2	9.05
NGB13469	Gråärt från Laholm,’Stäme’	Swedish landrace	44.0 (1.22)	hr	1	6.62	2	9.29
NGB103590	WBH3590 (Skararp)	Swedish landrace	60.2 (3.83)	HR	1	5.87	3	8.10
NGB14155	Skånsk gråärt	Swedish landrace	58.8 (4.15)	HR	1	6.66	3	8.73
NGB103518	WBH3518 (Tollestorp)	Swedish landrace	47.2 (4.09)	HR	1	6.14	1	7.55
NGB14153	Solberga	Swedish landrace	63.4 (1.95)	HR	1	6.19	2	7.85
NGB14639	Orust	Swedish landrace	77.0 (3.00)	HR	1	7.56	2	8.99
NGB13487	Östgöta gulärt	Swedish landrace	71.8 (1.92)	HR	1	6.2	1	8.53
NGB14638	Brattebräcka	Swedish landrace	87.2 (4.32)	HR	1	6.05	8	7.86
NGB17868	Väse	Swedish landrace	39.0 (2.00)	hr	1	8.28	1	8.23
NGB17881	Rättviks gråärt	Swedish landrace	54.8 (7.73)	HR	1	5.51	1	-
NGB103517	Jämtländsk grå	Swedish landrace	47.0 (4.18)	HR	1	6.41	5	8.31
NGB14642	Lit	Swedish landrace	49.3 (2.59)	HR	1	5.86	5	8.91
JIC1525	Prasia	European landrace	42.8 (2.39)	hr	1	6.07	6	7.42
JIC1778	Culdris	European landrace	56.8 (2.28)	HR	5	6.39	2	9.09
JIC1031	Germany	European landrace	50.0 (1.87)	HR	4	4.76	9	-
NGB102814	WBH2814	European landrace	67.4 (7.50)	HR	1	5.05	4	9.11
NGB17884	Vidzemes Tirgus	European landrace	32.4 (0.89)	HR	1	8.71	2	6.75
NGB17871	Baltikum	European landrace	54.4 (1.82)	hr	1	5.41	4	9.07
NGB17883	Papardes	European landrace	52.2 (1.92)	HR	1	7.28	1	8.16
NGB20117	Lollandske Rosiner	European landrace	47.0 (1.73)	hr	1	5.91	2	8.29
NGB20123	Errindler	European landrace	46.0 (1.00)	hr	1	7.22	1	8.14
NGB101997	Brioärt	Cultivar	43.6 (2.07)	hr	1	7.08	1	7.78
NGB10660	Capella	Cultivar	47.6 (0.55)	hr	1	5.75	4	8.75
NGB13138	Odalett	Cultivar	46.0 (1.58)	hr	1	6.45	1	8.56
NGB4018	Timo	Cultivar	43.2 (1.64)	hr	1	6.47	3	8.26
NGB103071	Rosakrone	Cultivar	52.8 (1.48)	HR	1	4.99	1	8.48
JIC1228	Wellensiek’s Dominant	Cultivar	68.0 (14.34)	HR	3	5.7	7	8.68
JIC1233	Murfet Line 60:E1	Cultivar	36.0 (0.82)	hr	2	8.22	3	10.00
NGB102027	WBH2027	Wild	74.6 (0.89)	HR	1	5.71	1	8.17
NGB102123	WBH2123	Wild	51.0 (2.00)	HR/hr	6	-	-	-
NGB103567	WBH3567	Wild	47.7 (4.73)	hr	-	6.51	1	9.46

^a^Mean (standard deviation)

^b^Cq value

Test cultivation of a subset of the accessions (all landraces and three cultivars) was carried out during the summer of 2011. Eight individuals of each accession were grown in the field under natural conditions in Gränna, Sweden (57°N, 15°E) and the number of days to first flower (DTF) was recorded. During 2012 five individuals of each accession (except four of JIC1228 and JIC1233 and three of NGB102123) were cultivated in a greenhouse in Linköping, Sweden. Plants were grown in standard commercial soil, and the cultivation temperature was set to a minimum of 16 °C. Light was provided artificially to create a day length of at least 16 h/day, to mimic natural conditions in Sweden during the growth season. The DTF, node at first flower (NAF), number of days to first mature pod (MAT) and total seed weight were recorded for all individuals. Young leaves were collected from single plants of each accession and dried with silica gel when not used directly for DNA extraction.

During 2013 single-seed descent lines of the same accessions (with the exception of NGB102123) were grown in a growth chamber with a 16 h light - 8 h dark regime. Five replicates of each accession (four of NGB102814) were grown with four plants per replicate in a split-plot design (in total 5 × 4 plants per accession). Cultivation temperature was set to 20 °C during light hours and 18 °C during dark hours. To ensure simultaneous germination, all seeds were scarified before sowing. When the fifth node of each individual appeared (according to [16]), the tip of the individual was harvested, frozen in liquid nitrogen and stored at -80° for later extraction of RNA.

### DNA sequence analysis

DNA was extracted using either the E.Z.N.A Plant DNA kit (Omega Bio-Tek, GA, USA) for dried leaves, or the DNeasy plant mini kit (Qiagen AB, Germany) for fresh leaves.

The three flowering time genes, *HR*, *LF* and *SN,* were sequenced in all accessions. *HR* was amplified using two sets of primers (ELF3-FF + ELF3-11R and ELF3-5 F + ELF3-RR) from [15]. *LF* was amplified with three sets of novel primers (LF5-F + LF5-R, LF6-F + LF6-R, LF7-F + LF7-R). *SN* was amplified using a single primer pair (LUX-5UTR-11 F + LUX11R). All primer sequences are given in Additional file 2.

Two different PCR reaction mixes were used as follows: For *HR* and *LF* the mix contained 2 μl of 10x buffer, 0.2 μl of dNTP (25.0 mM), 2 μl of each primer (1.0 μM), 0.2 μl of *Taq* DNA polymerase (5 U/μl, NE BioLabs), 2 μl of template DNA and 11.6 μl of nuclease free water. For *SN* the mix contained 2 μl of 10x Buffer for long PCR enzyme mix including 15 mM of MgCl_2_, 0.4 μl of dNTP mix (2 mM), 0.6 μl of each primer (10 μM), 0.1 μl of long PCR enzyme mix (5 U/μl Fermentas, Thermo Scientific), 0.4 μl of DMSO, 1 μl of template DNA and 15.9 μl of nuclease free water to bring the reaction mixture to 21 μl. PCR conditions were: initial denaturation at 94 °C for 1 min, then 37 cycles of 30 sec at 94 °C, 40 sec at 48 °C (*LF*) or 58 °C (*SN* and *HR*) and 80 sec at 68 °C. Final extension was for 10 min at 68 °C.

PCR products were cleaned with *Exo I* and FastAP Thermosensitive Alkaline Phosphatase (TAP; Thermo Scientific) prior to sequencing: 0.015 μl of *Exo I* enzyme, 0.15 μl of TAP and 5.835 μl of water per reaction volume of 15 μl. Samples were incubated at 37 °C for 30 min followed by 95 °C for 5 min. The homozygous nature of the inbreeding peas allowed direct sequencing to be carried out using the above mentioned primers with additional internal primers for *HR* and *SN* where the PCR products were too large to be sequenced in a single reaction (Additional file 2). Sequencing of PCR products was carried out by Macrogen Europe, the Netherlands, and sequences were obtained in both directions for the major parts of the three genes studied.

### RNA extraction and qPCR

The tissue sampled for RNA extraction (in total 164 tissue samples across 33 accessions) was ground with steel beads in 2 ml tubes in a Tissue Lyser (Qiagen AB, Germany). Frozen (-80 °C) adapter blocks with sample tubes were shaken for 1 minute at 25Hz which was repeated four times with freezing at -80 °C for 20 min between each shaking session. RNA was extracted from the pulverized tissue using the RNeasy Mini Kit (Qiagen AB, Germany) according to the manufacturer’s instructions. RNA concentrations were measured using NanoDrop ND-1000 (Thermo Scientific Inc., DE, USA). The qPCR primers for *LF*, *SN* and *TUB* genes are given in Additional file 2. *TUB* was used as a ‘housekeeping’ gene with a stable expression pattern against which the two other genes’ expressions were standardized.

The qPCR was performed in a one step protocol from the RNA samples by using QuantiFast SYBR Green RT-PCR (Qiagen AB, Germany) as described by the manufacturer. Samples with *LF* and *TUB* qPCR primers were run on the same plate with a negative reverse transcriptase control (qPCR performed without reverse transcriptase) for the *TUB* gene added to verify the absence of contaminating DNA. A blank sample for negative RNA control as well as two positive control samples for interplate calibration purposes were run with each primer pair on each plate. The *SN* gene qPCR was run separately along with the two positive control samples as well as a negative RNA sample with both *LF* and *TUB* qPCR primers.

### Data analysis

All statistical analyses and drawing of boxplots and scatterplots were performed using R [24], unless otherwise stated. Differences in means of DTF and total seed weight between landraces, cultivars and wild accessions were compared using the Welch two-sample *t*-test. Correlations between traits and environmental factors were calculated using Pearson’s product–moment correlation coefficient. The *psych* package [25] in R was used to obtain the descriptive statistics per group. Length of growth season at the site of origin of the accessions (GSO) was defined as the average number of days with a mean temperature of > +5 °C for the period of 1961–1990 (http://www.smhi.se/klimatdata/meteorologi/temperatur/vegetationsperiodens-langd-1.4076).

### DNA sequence analyses

DNA sequences were edited and aligned using BioEdit [26]. Sequence data are deposited in GenBank with accession numbers KU258421-85, KU285144, and KU311164-96. The DNAsp software [27] was used to calculate DNA polymorphism and diversity measures, as well as divergence between accession types. DNAsp was also used to obtain Tajima’s D with SNPs and indels, and Fu and Li’s D* and F* within the full data set and within different accession types.

### qPCR analyses and the effect of variation in genes and their expression to traits

Raw expression data from the Light Cycler 480 (Roche) was converted with LC480Conversion (http://www.hartfaalcentrum.nl/index.php?main=files&fileName=LC480Conversion.zip&description=LC480%20Conversion&sub=LC480Conversion) and then analysed through LinRegPCR [28] to obtain PCR efficiencies and Cq values. Next the *LF* and *SN* expression Cq data was calibrated separately across plates whereafter it was normalised against the *TUB* housekeeping gene using the GenEx software (MultiD Analyses, Sweden, available at http://www.multid.se/). Unpaired two-tailed t-tests for differences in mean expression between accessions were calculated using GenEx. The significance level corresponding to 0.05 after Bonferroni correction for multiple testing was set at 0.000095. The difference between *LF* and *SN* expression were tested using paired *t*-test in R [24].

Generalized linear model (GLM) analyses for estimating the effects of *HR* haplotype, *LF* and *SN* expression on the phenotypic and environmental variables were performed in R [24]. The GLM models analysed are detailed in Additional file 3. The response variables were DTF, MAT, NAF, seed weight and SN and LF expression. The explanatory variables were HR genotype, LF haplotype, SN haplotype, LF expression, SN expression, GSO, latitude and number of seeds. Interactions between some of the explanatory variables were included as detailed in Additional file 3. The data analysed were either the whole data set including all the accession types or Swedish landrace data only. This division was necessary since reliable information about GSO and latitude was only available for the Swedish landraces.

## Results

### Flowering time, time to maturity and yield

The number of days between sowing and flowering (Days To Flowering, DTF) ranged from 32 for four of the five individuals of the Latvian landrace NGB17884 to 92 for one of the individuals of the Swedish landrace NGB14154, with accession averages ranging from 32.4 to 87.2 days for NGB17884 and the Swedish landrace accession NGB14638, respectively (Table 1; Additional file 4). The early flowering reference accession JIC1233 had the second-most lowest DTF at 36.0 days. The late flowering reference accession JIC1228 flowered on average at 68 days which was faster than four of the Swedish landrace accessions and one of the wild accessions (Table 1; Additional file 4). The individuals within most accessions were very synchronous in DTF with a standard deviation of less than two for half of the accessions and with all but three accessions having a DTF standard deviation of less than 5 (Table 1).

**Fig. 1:**
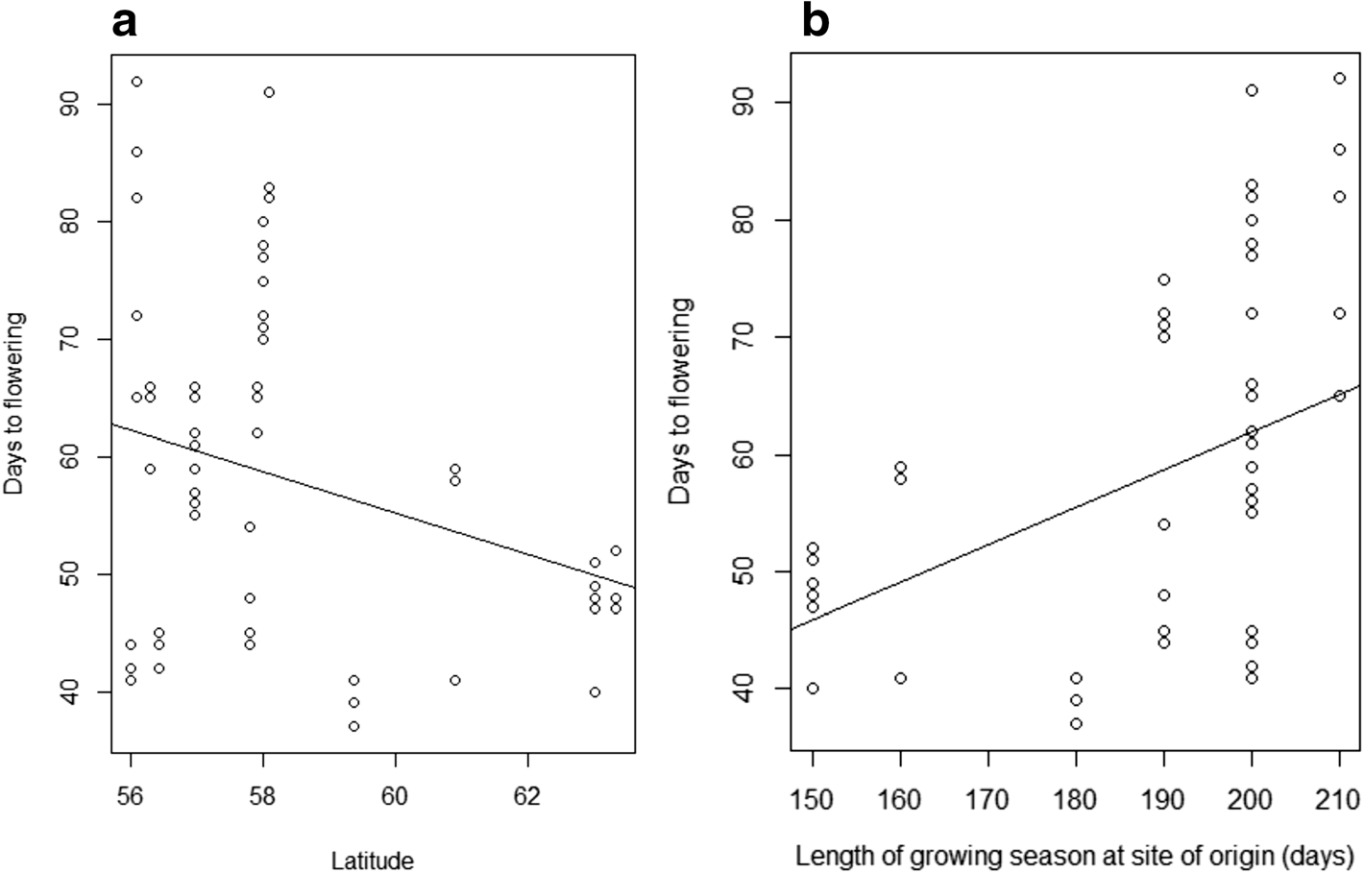
Scatterplots with regression lines showing the relationship of days to flowering (DTF) with (**a**) latitude and (**b**) the length of growing season, both at the site of origin for the Swedish landrace accessions

On average, Swedish landraces flowered significantly later than both European landraces and cultivars (58 vs. 50 and 48 days, respectively; both comparisons *p* < 0.001). Wild accessions had the most DTF on average (61 days), significantly more than both cultivars and European landraces (*p* < 0.05). Swedish landraces and wild accessions did, however, not differ in their DTF (*p* = 0.557). In general, the Swedish landraces had more variable flowering time than the other groups (Additional file 5). Among the Swedish landraces DTF was significantly negatively correlated with latitude (*r* = -0.29, *p* = 0.013, Fig. 1a, Additional file 6) and significantly positively correlated with GSO (*r* = 0.44, *p* < 0.001, Fig. 1b, Additional file 6) meaning that flowering was faster for accessions from higher latitudes and with shorter GSO.

Time to maturity after the plant started to flower (MAT) was negatively correlated with DTF (*r* = -0.48, *p* < 0.001; Additional files 6 and 7). In other words, the longer it took for a plant to flower, the faster the seeds matured, although the majority of variation in MAT was small and ranged between 30 and 35 days. The Swedish landraces and wild accessions matured significantly faster than cultivars (average difference of 2.5 and 3.2 days respectively, *p* < 0.0001 and 0.005, respectively). The differences between other accession types were not significant. The node at which the first flower was detected (NAF) was strongly correlated with DTF (*r* = 0.91, *p* < 0.001; Additional files 6 and 8), and therefore only DTF is reported below.

The Swedish landraces had on average a higher yield per plant (total seed weight, 9.97 g) compared to European landraces and cultivars (European landraces average 7.75, *p* < 0.0001; cultivars average 8.09, *p* < 0.05) whereas wild peas (average 6.92) and European landraces did not differ in total seed weight from the other accession types. The total seed weight was significantly correlated with DTF (*r* = 0.22, *p* < 0.01) (Additional files 1 and 6).

### *Sequence variation of* HR*,* LF *and* SN

**Fig. 2:**
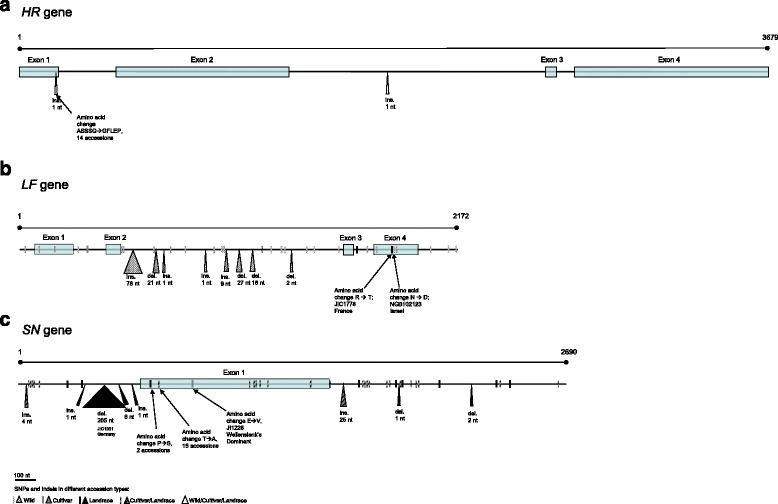
Line drawings of the sequenced regions of the (**a**) *HR,* (**b**) *LF* and (**c**) *SN* genes and their variation in the data set. Indels are represented by triangles and SNPs by vertical bars

DNA sequence was obtained for 3679 bp of the *HR* gene, spanning from Exon 1 to Exon 4, from all 34 accessions. The sequence variation in *HR* was very low and comprised of an indel in exon 1 (C5/C6) and another in intron 2 (T10/T11) (Fig. 2a, Additional file 9). The indel in exon 1 caused a previously known frame shift in the amino acid sequence starting from position 176 in our consensus sequence. This has been shown to result in an early termination codon after 21 amino acids and the difference between the early flowering *hr* and the late flowering *HR* allele [15]. Among our data set, 19 accessions had the *HR* allele (Haplotype 1: C5, T10) and 14 accessions the *hr* allele (Haplotype 2: C6, T11), with all but four of the Swedish landraces having the *HR* allele. The wild accession NGB102123 was heterozygous for both indels and was removed from further analyses.

The *LF* gene was sequenced for all the accessions in the study for 2172 bp spanning the whole length of the gene from 5’UTR to 3’UTR. The wild accession NGB103567 was highly heterozygous and was left out of further analyses. Among the sequences of the remaining accessions there were 29 SNPs and eight indels (all present only in the wild accession NGB102123) resulting in six haplotypes (Fig. 2b, Additional file 10). Haplotype 1 was identical to the [16] *LF* sequence (haplotype A), but neither of the other haplotypes corresponded to the haplotypes found by [16]. Haplotype 1 was present in 27 accessions and haplotype 3 in two with the remaining four haplotypes present in single accessions. The two reference accessions JIC1228 and JIC1233 differed from each other in two nucleotide positions. Two of the six SNPs located in the coding region caused an amino acid change within each of JIC1778 and NGB102123. The two changes were located at positions 127 (R - > T) and 129 (N - > D) of the protein sequence. They lie outside of the more conserved substrate binding sites and are not conserved across species. Only four SNPs of the 29 were found outside the wild accessions, and the Swedish landraces were all monomorphic with the exception of NGB17873 differing in a single SNP.

*SN* was by far the most variable of the three genes studied. The whole gene was sequenced for all accessions except NGB102123, along 2690 bp from the 5’UTR to the 3’UTR. In total 35 SNPs and nine indels were detected (Fig. 2c) resulting in nine haplotypes (Additional file 11) of which haplotypes 1 and 2 were found among 13 and seven accessions, respectively. The other seven haplotypes were found in one to four accessions each. Four of the nine haplotypes detected in this study shared the SNPs previously been described by [17]. Haplotypes 1, 4 and 7 in this study correspond to haplotypes 1, 20 and 3 in [17], respectively. The SNPs characterising haplotype 7 in [17] were found in haplotypes 2, 3 and 6 in this study. Liew et al. [17] found all of these changes from the reference sequence (haplotype 1 in their and this study) to be functionally non-significant with silent or conservative amino acid substitutions. In contrast with *LF*, the wild accessions shared haplotypes with landraces and cultivars. Nine SNPs were found within the coding region, three of which were non-synonymous changes present in a total of 18 accessions. All three non-synonymous changes occurred outside of the conserved parts of the protein, at positions 20 (*P* - > S), 31 (T - > A) and 86 (E - > V). The accession JIC1031 carried a 205 nucleotides long deletion that resulted in eight amino acids missing from the beginning of the coding sequence. This allele has not been described previously, and may well be a loss-of-function allele. However, we note that there is another in-frame start codon at position 30 in the amino acid sequence. The Swedish landraces had 26 polymorphic sites across the whole sequence with haplotype 5 being unique to the northernmost accessions but with no clear geographic distribution among the other four haplotypes present.

### Nucleotide diversity and Tajima’s D

DNA divergence (D_xy_) was calculated between all pairs of accession types for *LF* and *SN*, but not for *HR* where only indel variation was present (Table 2). Within *LF*, divergence between accession types was low between the Swedish and European landraces and cultivars (0.0001 – 0.0002) but higher between the wild accessions and the other accession types (0.0062 – 0.0064). In *SN* the divergence between accession types instead ranged more uniformly from 0.0024 between wild and Swedish landrace accessions to 0.0056 between cultivars and European landrace accessions.

**Table 2:** DNA divergence between accession types. D_xy_ for *SN* below the diagonal, D_xy_ for *LF* above diagonal

	Swedish landraces	European landraces	Cultivars	Wild
Swedish landraces	-	0.0001	0.0002	0.0062
European landraces	0.0051	-	0.0002	0.0063
Cultivars	0.0044	0.0056	-	0.0064
Wild	0.0024	0.0047	0.0031	-

Because there were only two wild accessions with sequence data for each gene, the wild accession type was omitted from further sequence analysis. Looking at all domesticated accessions, both haplotype and nucleotide diversity were higher for *SN* than for *HR* and *LF* (Table 3). Haplotype and nucleotide (Π_site_) diversities for the *HR* gene were similar for the different accession types as were nucleotide diversities for *LF*. In contrast the *LF* haplotype diversity, *SN* haplotype diversity and *SN* nucleotide diversity were all significantly lower for Swedish landraces than European landraces and cultivars (all comparisons *p* < 0.001 except *SN* nucleotide diversity for Swedish landraces vs cultivars: *p* < 0.05).

**Table 3:** Tests for selection and genetic diversity for *HR, LF* and *SN*. The wild accessions are not included in these analyses because there were too few sequences to obtain reliable results

	Tajima’s D		Fu and Li’s D*	Fu and Li’s F*	Haplotype diversity		Pi (site)	
	DNA	Indels	DNA	DNA	DNA^a^	Indels	DNA^a^	Indels
*HR*								
All data	na	2.080*	na	na	na	0.504	na	0.0003
Swedish landraces	na	0.953 ns	na	na	na	0.419	na	0.0002
European landraces	na	1.754 ns	na	na	na	0.556	na	0.0003
Cultivars	na	0.687 ns	na	na	na	0.476	na	0.0003
*LF*								
All data	−2.644***	−2.241**	−4.87*	−4.89*	0.333 (0.105)	0.061	0.0009 (0.00069)	0.00022
Swedish landraces	−1.159 ns	na	ns	ns	0.133 (0.112)	na	0.0002 (0.00005)	na
European landraces	−1.362 ns	na	ns	ns	0.417 (0.191)	na	0.0002 (0.00027)	na
Cultivars	−1.237 ns	na	ns	ns	0.524 (0.209)	na	0.0003 (0.00012)	na
*SN*								
All data	1.150 ns	−0.428 ns	1.57*	1.69*	0.742 (0.060)	0.722	0.0046 (0.00055)	0.00072
Swedish landraces	1.016 ns	0.429 ns	1.60*	1.65*	0.724 (0.084)	0.667	0.0037 (0.00066)	0.00064
European landraces	1.231 ns	1.152 ns	1.46*	1.60*	0.806 (0.089)	0.806	0.0062 (0.00088)	0.00076
Cultivars	0.800 ns	1.650 ns	ns	ns	0.905 (0.103)	0.571	0.0047 (0.00102)	0.00043

*ns* denotes *p* > 0.05, * denotes *p* < 0.05, ** denotes *p* < 0.01, *** denotes *p* < 0.001, na denotes not applicable

^a^ mean (standard deviation)

Tajima’s D was significantly positive for the total data set of *HR* (2.080, *p* < 0.05) although the power for the test based on a single indel is limited. In contrast Tajima’s D was significantly negative for the total data set of *LF* (DNA: -2.644, indels: -2.241; *p* < 0.001 and *p* < 0.01, respectively) (Table 3). Fu and Li’s D* and F* could not be calculated for *HR* due to the lack of variation, but were both significantly negative for the total data set of *LF* (Table 3). Neither accession type nor the total data set had significant Tajima’s D values for *SN*, but both Fu and Li’s D* and F* were significantly positive for the total data set and for Swedish and European landraces, but not for cultivars (Table 3).

### LF *and* SN *gene expression*

Gene expression of *LF* and *SN* was measured in 4 or 5 individuals from all accessions except NGB102123. In *SN*, the accession JIC1031, with a major deletion from the start of the protein, and the accession NGB17881, which persistently produced double melting peaks in all but one of the five replicates, were excluded from downstream analyses, resulting in 31 accessions analysed for *SN* and 33 for *LF* respectively. Negative controls verified the absence of DNA contamination and RNA cross-contamination.

With the exception of the accession NGB17884, *LF* was expressed significantly more (had lower Cq values) than *SN* in the target tissue of all accessions (*p* < 0.001, Table 1). Differences in expression between accessions were notably more pronounced in *LF* than in *SN*. From 528 pairwise t-tests between accessions for the *LF* gene expression 8 % were significant after Bonferroni correction while for the *SN* gene none were significant (Additional file 12). There were no significant differences in the expression levels of different haplotypes (*p* = 0.32 and 0.82 for *LF* and *SN* respectively), nor any significant differences in the *LF* or *SN* expression levels between the different accession types (all *p*-values > 0.05, Additional file 13). The two reference accessions differed, however, significantly from each other in *LF* expression (Additional file 12). Neither *LF* nor *SN* expression was correlated to either latitude or GSO (all *p* > 0.05).

### *Effects of* HR*,* LF *and* SN *variation on flowering time*

**Fig. 3:**
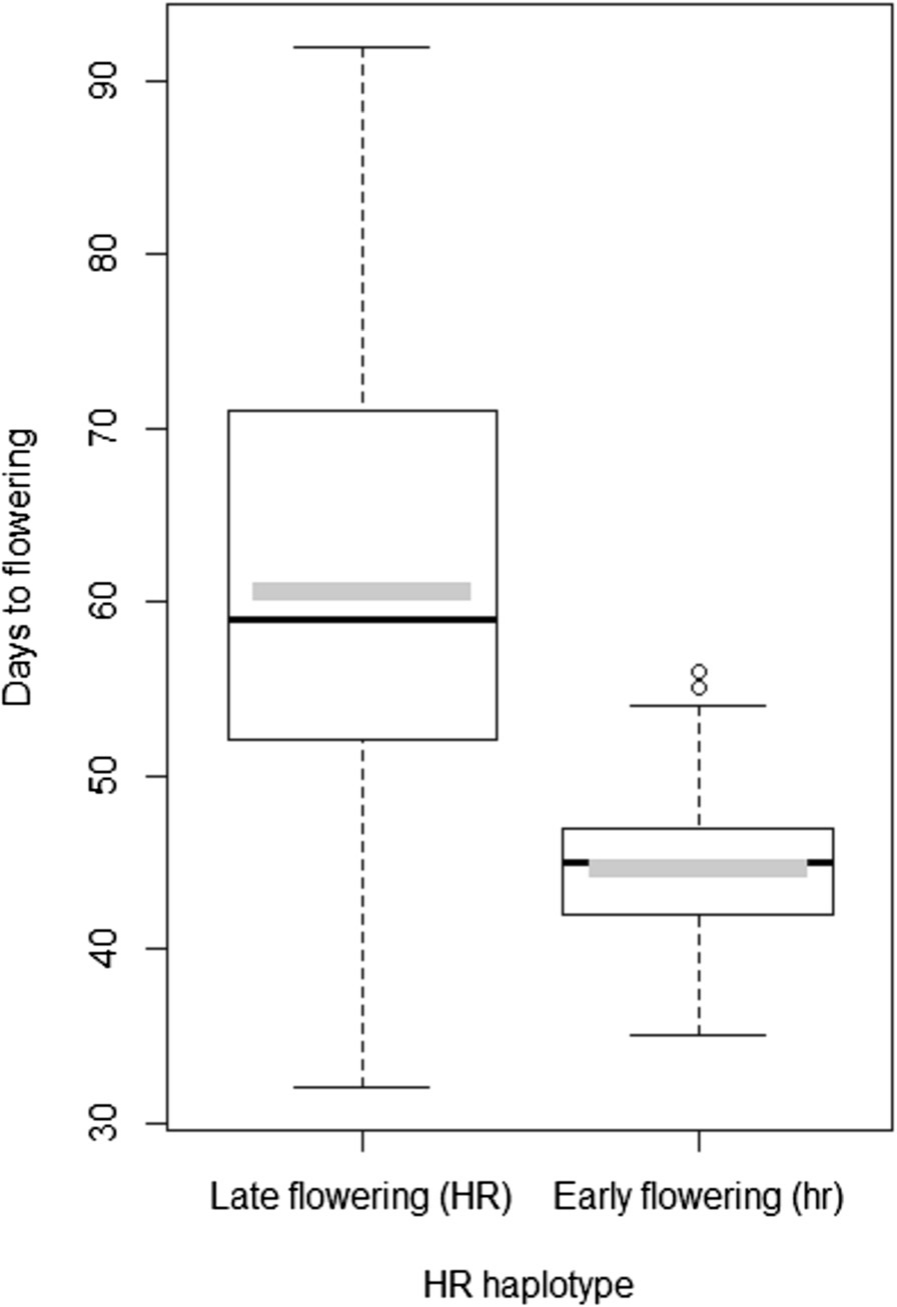
Boxplot showing days to flowering (DTF) for the two *HR* haplotypes. Mean and median values for DTF are shown with *grey* and *black* bars respectively

The two *HR* haplotypes had highly significantly different DTF (*t*-test, *p* < 0.001). *HR* (haplotype 1) was associated with late (average of 60.74 days) and *hr* (haplotype 2) with early (average of 44.81 days) flowering, with an average difference of 15.93 days (Fig. 3). Not all accessions, however, conformed to the DTF differences between the two haplotypes. The most striking exception was NGB17884, which had the *HR* haplotype, but flowered the earliest of all the accessions at 32.4 days (Table 1). NGB17884, however, had the lowest *LF* expression of all accessions (highest Cq value), which is most likely the cause of its early flowering.

**Fig. 4:**
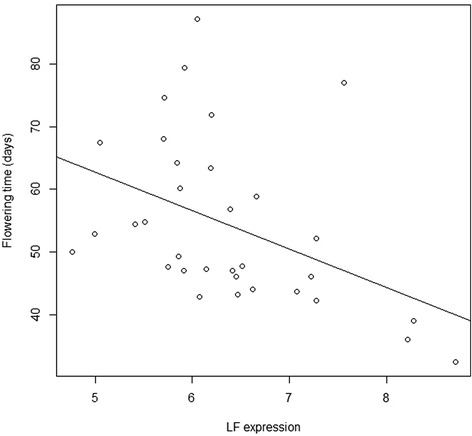
Scatterplot with a regression line showing the relationship between the average days to flowering (DTF) and the *LF* expression (Cq value)

There were no significant effects of *SN* haplotype, *SN* expression or *LF* haplotype on DTF (*p* = 0.7700, *p* = 0.3670 and *p* = 0.8390 respectively). The effect of *LF* expression on DTF was, however, significant (*p* < 0.05) and explained 18.6 % of the variation in DTF. A higher Cq value (lower *LF* expression) corresponded to a shorter flowering time (Fig. 4).

To analyse more complex relationships between factors with effect on DTF combined generalised linear model analyses were performed. The data sets used, the various models and their significances are detailed in Additional file 3. Among the Swedish landrace accessions *HR* haplotype together with GSO had a significant effect on DTF (*p* < 0.001 and *p* < 0.01; 23.68 and 0.41 days, respectively) and together they explained 49.6 % of the variation in DTF. There was no significant effect of *LF* or *SN* expression together with GSO detected for DTF. In combination with *HR* haplotype (DTF ~ LF + HR), the *LF* expression effect on DTF was close to, but not significant (*p* = 0.0593).

## Discussion

### *The effect of* HR*,* LF *and* SN *on flowering time in pea*

Our results confirm many of the previously found correlations between genetic diversity in the three studied genes and phenotypic diversity with regards to flowering time. In our sample of accessions from across Europe, including wild accessions, we found limited genetic variation in *HR*. We detected only two of the 15 haplotypes reported by Weller et al. [15] from a study of 110 wild and domesticated pea accessions from around the world. These two haplotypes have been shown to be the most common ones among European accessions [15]. Although the plants were cultivated under LD conditions, the two haplotypes allowed us to confirm the previously reported effect of *HR* on DTF with earlier flowering among individuals carrying the *hr* allele [15]. The accessions carrying the *HR* allele did, however, display a larger variation in flowering time and several accessions with a DTF smaller than 50 carried the *HR* allele (Fig. 3). For example the Latvian landrace NGB17884, the earliest flowering of our accessions, carries the *HR* allele. Similar results have previously been reported by Weller et al. [15] who found that several early flowering accessions carried the *HR* allele. Thus, even though the *HR* haplotype is a major influencer of flowering time in pea, it is clear other genes can also have an important impact on flowering time.

Weller et al. [15] commented that their single marker tests failed to provide evidence for significant effects of other known flowering loci, including *LF*, in a cross between the line NGB5839 (a derivative of the Swedish cultivar ‘Torsdag’) and the wild line JIC1794 (*P. elatius* var. *humile*). However, other studies have shown that recessive *lf* alleles can confer earlier flowering independently of the *HR* genotype [29]. We could confirm both the absence of correlation between coding sequence variants of *LF* and DTF and the correlation between *LF* expression and DTF found by Foucher et al. [16] with the early and late flowering reference accessions having low and high *LF* expression respectively. In our material a one-unit decrease in the Cq value of *LF* was associated with a 6.1 days delay in flowering time. Relatively early flowering accessions with high *LF* expression (low Cq values) and late flowering accessions with a low *LF* expression were found, but the three accessions with the lowest *LF* expression (NGB17868, NGB17884 and JIC1233) were also the earliest flowering (<40 days).

Genetic variation at the *SN* locus has previously been shown to affect flowering time and accessions carrying loss-of-function alleles of the gene are known to be early flowering [17]. None of the previously described mutations found in this study were loss-of function mutations and neither were they functionally significant according to Liew et al. [17]. Surprisingly, our qPCR showed that JIC1031, in spite of carrying a large deletion at the beginning of the gene, still expressed *SN*, which could explain its medium length flowering time and the absence of the early phenotype typically detected in *sn* mutants [17]. In fact, we were unable to show any significant effect of *SN* on DTF, neither from sequence variants, not from its expression. Also, although *HR* and *SN* are known to interact and regulate each other [17], this interaction may be complex, and we could find no connection between *SN* and *HR* in the present study. The expression of *SN* varies over the day [17], and although care was taken to harvest the samples at approximately the same time of day (~3 h after the beginning of light) differences in the sampling time or sampling at a suboptimal time point could have masked an effect of *SN*. Additionally DTF was only measured under LD condition and an effect of *SN* expression has so far only been described under SD conditions [17].

### Flowering time and local adaptation in Swedish landrace pea

Sweden stretches for some 1600 km from below 56° N to just above 69° N. Although pea cultivation is not possible over the whole country, the length of the growth season in the parts of the country where peas are being or have been cultivated still ranges from approximately 140 to 220 days. Thus, Sweden offers a range of local climatic conditions for peas to adapt during the millennia of pea cultivation, and a rapid flowering and a quick maturation is necessary in the regions with the shortest growth seasons.

The Swedish pea landraces showed evidence of phenotypic adaptation to the varying cultivation climate occurring across Sweden although the connection was not entirely straightforward. The large variation in DTF present among Swedish landraces was significantly correlated with latitude. Although the main range of Sweden is in the north – south direction, latitude is not the sole determinant of climate in the country and consequently GSO was more strongly correlated with DTF than latitude. In regions with a short growth season natural selection has favoured rapidly flowering genotypes. The variation in flowering time was much larger in more southern parts of Sweden and areas with a longer GSO, partially obscuring the correlation between DTF and latitude and GSO. In the absence of strong natural selection for rapid flowering a longer growth phase before flowering has been possible. A delayed flowering could lead to a larger harvest, and in our material, a longer DTF was correlated with a higher total seed weight of the plant. It is possible that in some accession artificial selection by the farmers for larger harvest has resulted in a longer growth phase resulting in a higher yield. Additionally, the vegetative parts of the plant were considered valuable fodder in the past [8], and a delayed flowering, leading to larger production of total biomass, may also have been sought by some farmers. We note that accessions from regions with a longer growing season have more varied flowering times indicating that in these regions climate allowed for differentiation into types with different properties, something that was not possible in harsher climates.

The data on DTF used in this study was obtained from cultivation in a controlled greenhouse climate. Under field conditions the DTF of an overlapping set of accessions (including all landraces from this study and three out of the seven cultivars) showed a strong and significant correlation (*r* = 0.65, *p* = 0.0002) to the DTF obtained in our greenhouse cultivation (Additional file 14). This correlation is comparable with Burstin et al. [30] where the correlation of DTF between two different field trials was 0.79. Thus we consider the greenhouse data for DTF to be a reasonably good indicator for the DTF under field conditions.

### Variation in flowering time genes in Swedish landrace peas

In spite of the correlation between DTF and latitude and GSO, the connection between latitude and GSO and genetic and expression diversity of the three genes studied is less clear. The lack of clear geographic distribution of *LF* and *SN* haplotypes and *SN* expression is not unexpected considering that no effect of them on DTF could be found. More surprising is the lack of geographical distribution at the *HR* locus. Only three Swedish landrace accessions had the early flowering *hr* allele but neither originated from particularly far north. For example, the three northernmost accessions all had the late flowering *HR* allele, but in general flowered earlier than *HR* accessions origination from further south.

The expression of *LF* did not show any significant correlation with latitude or GSO in the Swedish landrace accessions. This may seem surprising given the correlation of *LF* expression and DTF we found. We note, however, the wide diversity in DTF present in lower latitudes and regions with longer GSO. It is possible that a significant correlation with *LF* expression could have been detected in a larger data set or a data set where DTF was more strongly correlated with latitude and GSO.

Flowering time is a complex trait controlled by multiple genes and flowering time will rarely be determined by single SNPs (but see [15, 21]). Here we have only studied three of the genes influencing flowering time in pea. Further studies of loci such as *DIE NEUTRALIS* (*DNE* [31]), *GIGAS/FTa* and *FTb* [32], known to influence the transition to flowering, may well show their importance for local adaptation of flowering time in Swedish landraces. In addition, control of gene expression can be complex with trans and cis regulation feedback loops. The final expression of flowering time genes will depend on gene interactions but also plasticity. Most likely flowering time adaptation is the consequence of complex interactions between a number of genes, similar to the situation suggested for Nordic barley landraces [22].

### Evolution of flowering time in domestic pea

From our limited sample of wild pea it was clear that they displayed varied DTF, and were in general more phenotypically and genetically diverse than the other accession types. High genetic diversity in wild peas has previously been shown with different neutral genetic markers [2, 30, 33] and a higher genetic diversity in wild progenitors than domesticated plants is congruent with theoretical predictions about the domestication process and empirical comparisons of wild and domesticated plants (e.g. [34]). Burstin et al. [30] pointed out that even though the variability is larger in wild peas, it does not mean that the domesticated gene pool is lacking in variation. Instead they concluded that diversity across pea varieties has probably been maintained through breeding for diverse end uses such as fodder crops and garden peas. Different climatic conditions may also have been a force preserving genetic and phenotypic diversity in pea.

In spite of the known role of *HR*, *LF* and *SN* in determining flowering time we find limited support for selection for local adaptation having acted upon them. Only *LF* displayed the negative test values indicative of directional selection when the full dataset is analysed. For the different accession types Fu and Li’s statistics (but not Tajima’s D) instead indicated balancing selection acting on *SN*. Considering the range of flowering times observed in both wild and domestic accessions it is possible that different alleles have been selected simultaneously or that soft rather than hard selective sweeps have been responsible for flowering time adaptation.

The development of commercially available pea cultivars in Sweden resulted in a reduction in DTF (under LD conditions studied here and prevailing during pea cultivation in Sweden). The selection of genetically homogeneous cultivars also resulted in a reduced within-accession variability both in flowering time and in seed production traits. Four of the five Swedish cultivars carried *hr* alleles, and it is possible that the early flowering *hr* allele has been important in the breeding of rapidly flowering cultivars as the demand for biomass for fodder declined. It is also possible that active breeding for shorter, sturdier plants resulted in cultivars flowering at fewer nodes, i.e. at lower DTF. In contrast MAT was longer for cultivars than the Swedish landraces they were at least partially developed from. A longer MAT allows for longer grain filling and may have been a criterion selected for during the development of pea cultivars. Today, pea is typically grown in the south of Sweden and in 2014 no harvests of pea were reported from areas above the 60^th^ parallel (Statistics Sweden, 2015, http://www.scb.se/). This, however, seems not to be a consequence of a long DTF in commercial cultivars as some of the commercial cultivars flowered as rapidly as landrace accessions from north of the 60^th^ parallel. Investigation on other adaptation traits such as frost tolerance and drought resistance would allow more complex aspects of local adaptation in this material to be explored.

## Conclusions

From our study of the flowering time of wild peas, European and Swedish landraces and commercial pea varieties and the genetic diversity of three flowering time genes we find that Swedish landraces have adapted locally to differences in growth season. We can further confirm several previously published effects of sequence and expression diversity on flowering time from the genes *HR*, *LF* and *SN*. The genetic causes of the flowering time adaptation in Swedish landraces are more obscure and seem to involve the studied genes as well as interactions between them and likely other genes not investigated in this study.

## Abbreviations

DTF, days to flowering; GSO, length of growing season at site of origin; SD, short day; LD, long day; DTF, days to first flower; NAF, node at first flower; MAT, days to first mature pod; GLM, generalized linear model.

## Additional files

Additional file 1: Accessions used in the study, their origin and data on flowering and seed production. (XLSX 51 kb) Additional file 2: Primers used in the study. (PDF 16 kb) Additional file 3: GLM models analysed, their significance and the data sets used. (XLS 22 kb) Additional file 4: Boxplot showing days to flowering (DTF) within each accession. Mean and median values for DTF are shown with red and black bars respectively. (PDF 69 kb) Additional file 5: Boxplot showing days to flowering (DTF) within each accession type. Mean and median values for DTF are shown with red and black bars respectively. (PDF 44 kb) Additional file 6: Pearson correlation coefficients (including significances) between the measured traits, haplotypes and expression levels. Correlations with latitude and GSO are done with Swedish landraces only while the rest of the correlations include the whole data set. (XLS 21 kb) Additional file 7: Scatterplot showing the relationship of days to flowering (DTF) and time to maturity after flowering (MAT). Accession types are shown with different symbols. (PDF 47 kb) Additional file 8: Scatterplot showing the relationship of days to flowering (DTF) and the node at first flower (NAF). Accession types are shown with different symbols. (PDF 43 kb) Additional file 9: Variation in the *HR* gene. (XLSX 11 kb) Additional file 10: Variation in the *LF* gene. (XLSX 16 kb) Additional file 11: Variation in the *SN* gene. (XLSX 16 kb) Additional file 12:
*P*-values of Unpaired *t*-test for gene expression levels between pairs of accessions. (XLSX 48 kb) Additional file 13:
*P*-values for Welch two sample t-tests between different accession types and expression levels for the *LF* and *SN* gene. (XLSX 33 kb) Additional file 14: Scatterplot with a regression line of the relationship between DTF measurements in the greenhouse and field with overlapping accessions between the two studies. (PDF 40 kb)
