# Supplementary material for: Flowering time adaption in Swedish landrace pea (Pisum sativum L.)
Source: BMC Genet. 2016 Aug 12;17:117. doi: 10.1186/s12863-016-0424-z (PMC4983087; doi:10.1186/s12863-016-0424-z)
Supplement: Additional file 2: — Primers used in the study. (PDF 16 kb) [file 12863_2016_424_MOESM2_ESM.pdf]

Additional file 2. Primers used in the study

Primer name	primer sequence (5'-3')	gene	used for	reference
ELF3-FF	GTTTAGAGTTTAGGATAGAAAAGGGGTAGG	HR	PCR	Weller et al, 2012
ELF3-11R	GCAATTTCTTTTCTGGCTTTCC	HR	PCR	Weller et al, 2012
ELF3-5F	ACCAGTCCAACCCAGGCTAT	HR	PCR	Weller et al, 2012
ELF3-RR	GATCCTCCATGTCAATATACACCACTAC	HR	PCR	Weller et al, 2012
LF5-F	ACACCGTATAGCTCTCGT	LF	PCR	This study
LF5-R	CCTACCACTTTAAATGATTATTACC	LF	PCR	This study
LF6-F	TGTTTCTGAGTTTCTAGTAGTGAGT	LF	PCR	This study
LF6-R	CCTGGAATGTCTGTCACCATCCTA	LF	PCR	This study
LF7-F	GCTAGGTTTAAGCGATTTCATAGCC	LF	PCR	This study
LF7-R	TTGAGCATTACCACACCA	LF	PCR	This study
LUX-5UTR-11F	GAGATTAAGTCGCAGAAACCAA	SN	PCR	Also used in Liew et al, 2014
LUX-11R	CTCAAGAATCAGTTTGTGCA	SN	PCR	Also used in Liew et al, 2014
LFQF2	TTCCAGGGACAACAGATTCC	LF	qPCR	This study
LFQR2	CAGGGACACCAAGGTCATTT	LF	qPCR	This study
LUXQ2F	TAGGGATTGGCCTCCTCATC	SN	qPCR	This study
LUXQ2R	TGGATGAAGTCACAATCAACA	SN	qPCR	This study
PeabtubF1	GCTCCCAGCAGTACAGGACTCT	TUB	qPCR	This study
PeabtubR1	TGGCATCCCACATTTGTTGA	TUB	qPCR	This study
ELF3-4R	GTTTCCCAGCCTGACGAAT	HR	sequencing	Weller et al, 2012
ELF3-7F	TGTTTGCAGTCCAAGTGTTTG	HR	sequencing	Weller et al, 2012
ELF3-7R	CGATCCGGCAATTAGTTGTT	HR	sequencing	Weller et al, 2012
LUX-4R	CAGCGTCGTTTCTGTTCTAACC	SN	sequencing	Also used in Liew et al, 2014
LUX-7R	GTTGGAAAGACCTTGCATCC	SN	sequencing	Also used in Liew et al, 2014
LUX-9F	CGGGACAAATCACCAAACG	SN	sequencing	Also used in Liew et al, 2014
LUX-Q1F	TTCTCACCCTCACATGTCTCC	SN	sequencing	Also used in Liew et al, 2014
