# Supplementary material for: Flowering time adaption in Swedish landrace pea (Pisum sativum L.)
Source: BMC Genet. 2016 Aug 12;17:117. doi: 10.1186/s12863-016-0424-z (PMC4983087; doi:10.1186/s12863-016-0424-z)

Additional file 4. Boxplot showing days to flowering (DTF) within each accession. Mean and median values for DTF are shown with red and black bars respectively.

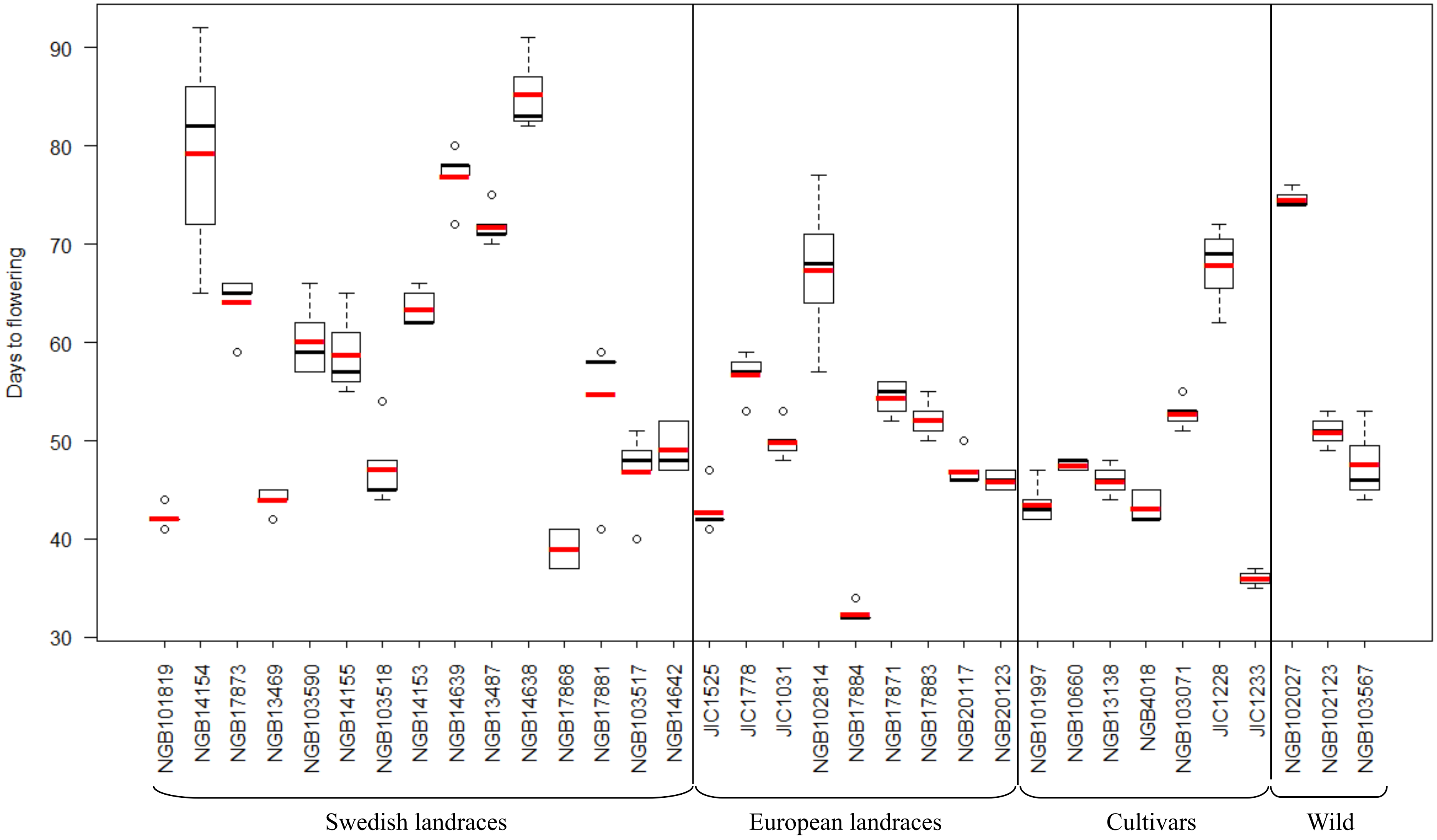

Supplement: Additional file 4: — Boxplot showing days to flowering (DTF) within each accession. Mean and median values for DTF are shown with red and black bars respectively. (PDF 69 kb) [file 12863_2016_424_MOESM4_ESM.pdf]
