# Supplementary material for: Flowering time adaption in Swedish landrace pea (Pisum sativum L.)
Source: BMC Genet. 2016 Aug 12;17:117. doi: 10.1186/s12863-016-0424-z (PMC4983087; doi:10.1186/s12863-016-0424-z)

Additional file 8. Scatterplot showing the relationship of days to flowering (DTF) and the node at first flower (NAF). Accession types are shown with different symbols.

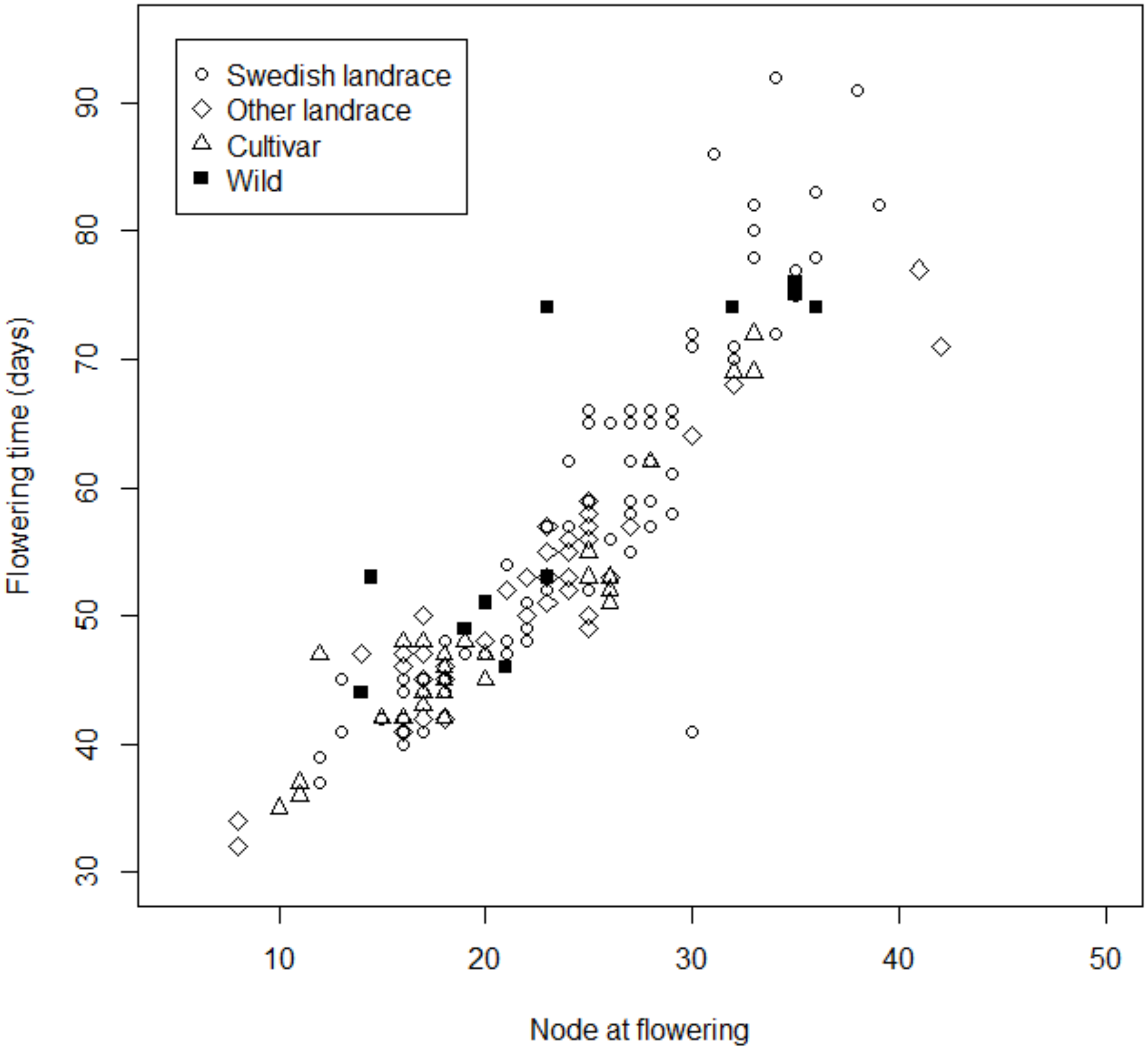

Supplement: Additional file 8: — Scatterplot showing the relationship of days to flowering (DTF) and the node at first flower (NAF). Accession types are shown with different symbols. (PDF 43 kb) [file 12863_2016_424_MOESM8_ESM.pdf]
