# Supplementary material for: Flowering time adaption in Swedish landrace pea (Pisum sativum L.)
Source: BMC Genet. 2016 Aug 12;17:117. doi: 10.1186/s12863-016-0424-z (PMC4983087; doi:10.1186/s12863-016-0424-z)

Additional file 14. Scatterplot with a regression line of the relationship between DTF measurements in the greenhouse and field with overlapping accessions between the two studies.

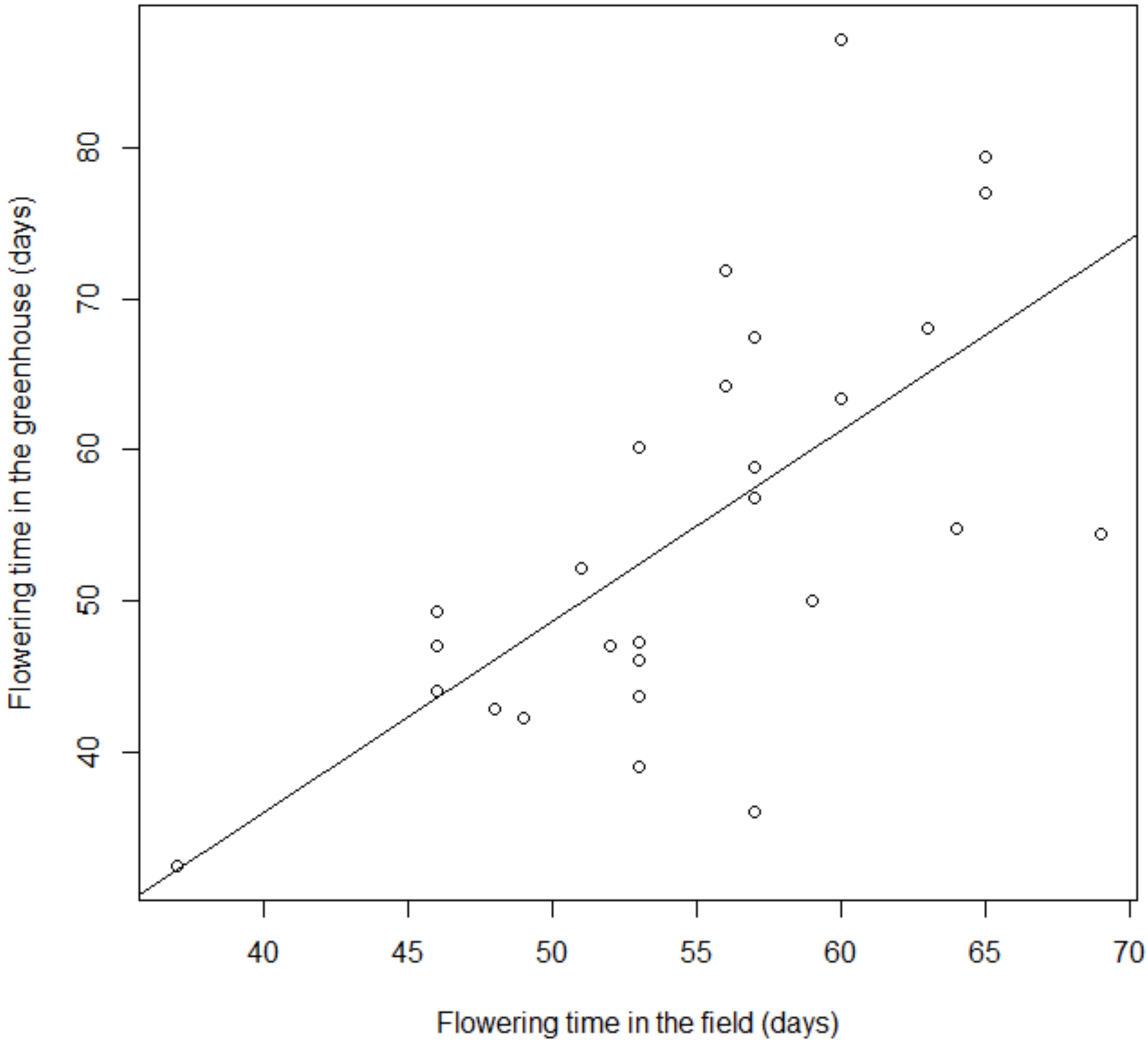

Supplement: Additional file 14: — Scatterplot with a regression line of the relationship between DTF measurements in the greenhouse and field with overlapping accessions between the two studies. (PDF 40 kb) [file 12863_2016_424_MOESM14_ESM.pdf]
